# Systemic thrombolysis in the management of pump thrombosis in patients with left ventricular assist devices

**DOI:** 10.3389/fcvm.2022.969766

**Published:** 2022-10-13

**Authors:** Kirsten A. Kortekaas, Paul L. den Exter, Saskia L. M. A. Beeres, Meindert Palmen, J. Wouter Jukema, Menno V. Huisman, Laurens F. Tops

**Affiliations:** ^1^Department of Cardiology, Leiden University Medical Center, Leiden, Netherlands; ^2^Department of Thrombosis and Hemostasis, Leiden University Medical Center, Leiden, Netherlands; ^3^Department of Cardiothoracic Surgery, Leiden University Medical Center, Leiden, Netherlands

**Keywords:** bleeding complication, left ventricular assist device (LVAD), pump thrombosis (PT), thrombolysis, thrombotic complication

## Abstract

Left ventricular assist device (LVAD) implantation as destination therapy (DT) is a valuable treatment option in patients with end-stage heart failure ineligible for heart transplant. However, this therapy can be complicated by life-threatening pump thrombosis (PT). This case series reports our single-center experience with a structured systemic thrombolysis protocol in case of PT. Consecutive patients undergoing DT LVAD (HVAD, Medtronic, Framingham, MA) implantation between 2010 and April 2021 at our institution were reviewed and those with PT identified. Clinical, laboratory and LVAD specific data were collected and analyzed retrospectively. All patients with PT were treated with systemic thrombolysis according to a structured bedside protocol. Treatment was defined successful if a patient was alive at 30 days follow-up and free of recurrent PT, stroke or device exchange. Fourteen out of 94 patients experienced a PT after LVAD implantation (11%). Systemic thrombolysis was successful in 10 of 14 patients (71%) at 30 days. Two patients died within 30 days due to a hemothorax and multi-organ failure. In three patients treatment was complicated by a major bleeding; twice a hemothorax (one fatal) and one right calf bleeding. No intracerebral hemorrhage was observed. Three patients experienced a thrombotic complication within 30 days; all recurrent PT. Eleven of the 14 DT patients were discharged home after a limited hospital stay after thrombolysis (average of 11 days). In conclusion, systemic thrombolysis may be a reasonable option for life-threatening PT in this vulnerable DT group in whom device exchange is often impossible due to comorbidity.

## Introduction

Left ventricular assist device (LVAD) therapy is a viable option for patients with advanced heart failure (HF). Device innovation and a subsequent reduction in adverse events has resulted in a more widespread use of LVADs as destination therapy (DT). However, unintended device-related adverse events still exist, including pump thrombosis (PT) which may negatively affect clinical outcomes.

Patients require anticoagulant therapy because they face a significant risk of thromboembolic complications due to the presence of the artificial surfaces of the pump and the modified fluid dynamic pattern of the blood accompanied by shear forces (1). If PT occurs, the available treatment options are heart transplant, device exchange, intensification of anticoagulant treatment and/or systemic thrombolysis. Heart transplant is not an option in DT patients and (urgent) pump exchange implies major surgery in a vulnerable patient population with considerable surgical risk (2), leaving systemic thrombolysis as the main treatment option (3). This study aims to evaluate our experience in treating DT LVAD patients with PT by means of a structured systemic thrombolysis protocol.

## Materials and methods

The study population consisted of all patients with advanced HF undergoing LVAD implantation (HVAD, Medtronic, Framingham, MA) as DT from November 2010 until April 2021. This single-center retrospective analysis of a prospectively collected cohort was reviewed by the local medical ethics committee (G20.182) who waived the need for official approval according to the Medical Research Involving Human Subjects Act.

Clinical, laboratory and LVAD specific data were collected and analyzed retrospectively from the patient information systems. Baseline characteristics, including laboratory values and medication use (antiplatelet regimen with clopidogrel preferred in our institution), were collected the day before PT (or most recent if not available). Pump thrombosis was defined as ≥2 signs or symptoms of PT in combination with an accompanying intervention such as intensified treatment with anti-coagulation, intravenous thrombolytics or pump replacement (4). The following signs and symptoms were considered suggestive of PT: (1) presence of biochemical signs of hemolysis, (2) worsening of HF and (3) abnormal pump parameters (4). All patients presenting with PT were initially treated with systemic thrombolysis as the first-line treatment, given the fact that all patients were no candidate for heart transplantation (our institution is a non-cardiac transplant center) and operative risk for pump exchange was deemed too high after a case by case discussion within our dedicated LVAD team including cardiologists, thrombosis and haemostasis experts, and cardiothoracic surgeons. In principle, the systemic thrombolysis protocol for all patients comprised of 10 mg alteplase iv (bolus) followed by 90 mg in 2 hours (structured bedside protocol). In the absence of guidelines specifically providing guidance for thrombolysis in LVAD pump thrombosis, we choose to adhere to the high-risk pulmonary embolism protocol with deemed maximal fibrinolytic effect. All patients were admitted to the intensive care unit or cardiac care unit and vital signs were continuously monitored in addition to LVAD parameters, neurological signs and symptoms, bleeding related complications, and hemoglobin tests. In case of history of intracerebral hemorrhage or other bleeding related risk factors a tailored approach was applied after consultation of thrombosis/hemostasis experts. Bleeding complications were defined as major or minor according to the International Society of Thrombosis and Haemostasis (ISTH) criteria (5). After administration of alteplase, thrombolysis was deemed successful if the LVAD pump parameters normalized and markers for hemolysis improved. In accordance with previous studies, treatment success was defined successful if a patient was alive at 30 days follow-up and free of recurrent PT, stroke or device exchange (6, 7).

## Results

Fourteen out of 94 patients experienced PT after LVAD implantation (11%). Table 1 depicts the baseline characteristics of these patients. All patients used a combination of phenprocoumon and antiplatelet therapy except for two patients. One patient (patient 5) was recently diagnosed with a intracerebral hemorrhage and received low molecular weight heparin (LMWH) in a therapeutic dosage. The second patient (patient 6) was still at the ICU due to a complicated clinical course after LVAD implantation, and received heparin guided by the aPTT (target 60–80 s). The heparin dosage was lowered for a tracheostomy and 2 days thereafter a PT was diagnosed (lowest aPTT value: 48 s). Of note, patient 12, with a history of atrial fibrillation and subdural hematoma after LVAD implantation, used phenprocoumon in combination with aspirin.

**Table 1:** Baseline characteristics and laboratory values before pump thrombosis.

**Patient no**.	**1**	**2**	**3**	**4**	**5**	**6**	**7**	**8**	**9**	**10**	**11**	**12**	**13**	**14**
Gender	Male	Male	Female	Male	Male	Male	Male	Female	Male	Male	Female	Male	Female	Male
Age (years)	67	65	39	60	64	67	56	69	62	68	59	70	59	71
BMI (kg/m^2^)	27	25	29	23	24	26	27	26	35	22	29	25	23	26
Etiology of HF	Ischemic	Ischemic	Non-ischemic	Ischemic	Non-ischemic	Ischemic	Ischemic	Non-ischemic	Ischemic	Non-ischemic	Ischemic	Ischemic	Ischemic	Non-ischemic
Atrial fibrillation	No	Yes	No	No	No	Yes	No	Yes	Yes	Yes	No	Yes	Yes	Yes
Creatinin (umol/L)	94	119	98	363	156	150	103	112	130	160	160	242	207	156
BUN (mmol/L)	5,3	4,1	6,5	13,9	9,8	39	10,7	10,3	6,8	18,9	10,7	27,5	12,4	16,0
Hemoglobin (mmol/L)	8,2	7,4	7,6	7,1	8,2	5,4	6,2	6,4	9,7	5,4	6,9	6,1	5,2	7,2
Hematocrit (%)	0,43	0,38	0,39	0,35	0,42	0,28	0,31	0,33	0,44	0,28	0,32	0,30	0,26	0,36
Phenprocoumon	Yes	Yes	Yes	Yes	No	No	Yes	Yes	Yes	Yes	Yes	Yes	Yes	Yes
Clopidogrel	Yes	Yes	Yes	Yes	No	Yes	Yes	Yes	Yes	Yes	Yes	No	No	Yes
Prasugrel	No	No	No	No	No	No	No	No	No	No	No	No	Yes	No

Patient data at baseline. All patients used phenprocoumon and antiplatelet therapy except patient 5 and patient 6. Patient 12 used phenprocoumon in combination with aspirin. Patient 3 was a 39 year-old female implanted as destination therapy because she was not eligible for transplant due to malignancy.

BMI, body mass index; BUN, blood urea nitrogen; HF, heart failure.

Table 2 shows the risk factors for PT and laboratory values used for the diagnosis of PT. Three patients experienced a recent bleeding (intracerebral hemorrhage in 2 patients, gastro-intestinal blood loss in 1 patient). In five patients oral anticoagulation was discontinued/lowered for invasive procedures or after intracerebral hemorrhage. After systemic thrombolysis, LVAD parameters normalized in all patients except one showing a persistent high flow (patient 8). This patient received three doses of systemic thrombolysis within 3 days. Extracorporeal membrane oxygenation as bridge to device exchange was used but the patient passed away due to multi organ failure. Also in patient 14 device exchange was considered, however, the patient refused this option and therefore received several doses of systemic thrombolysis.

**Table 2:** Risk factors for pump thrombosis, laboratory values at the time of pump thrombosis and LVAD values peri-intervention.

**Patient no**.	**1**	**2**	**3**	**4**	**5**	**6**	**7**	**8**	**9**	**10**	**11**	**12**	**13**	**14**
Recent bleeding	No	No	No	No	Yes	No	No	No	No	No	Yes	No	Yes	No
Recent inhibition OAC	No	Yes	No	No	Yes	Yes	No	No	No	No	Yes	No	No	Yes
MAP (mmHg)	87	68	62	78	79	70	65	50	81	75	100	108	71	77
Time between implantation and 1st PT (days)	335	66	1774	74	982	22	8	288	651	337	559	300	804	445
INR	2,1	2,5	4,3	2,9	1,1	1,2	3,9	2,7	2,2	2,2	2,3	2,5	3,1	2,6
LDH (U/L)	721	756	793	3424	2700	650	535	838	N/A	N/A	905	241	933	N/A
Total bilirubin (umol/L)	21	29	67	35	22	34	12	30	28	24	16	N/A	16	28
Conjugated bilirubin (umol/L)	7	15	22	< 1	N/A	18	N/A	18	N/A	6	N/A	N/A	N/A	N/A
Haptoglobin (g/L)	N/A	< 0,1	< 0,1	N/A	< 0,1	0,3	3,7	< 0,1	< 0,1	< 0,1	< 0,1	1,4	0,2	N/A
Free hemoglobin (umol/L)	N/A	9	283	N/A	N/A	2	1	7	83	61	50	N/A	42	344
**Baseline**														
LVAD flow (L/min)	4.7	5,7	2,5	4,2	3,1	5,1	4,6	4,4	5,0	3,3	3,2	3,3	4,1	3,9
LVAD power (watt)	3.3	2,8	2,7	2,7	3,0	3,5	3,1	3,3	4,2	3,2	3,3	3,1	3,5	3,1
LVAD speed (rpm)	2400	2300	2360	2340	2400	2560	2360	2480	2500	2400	2400	2400	2500	2400
**Pre intervention**														
LVAD flow (L/min)	7.1	9,5	7,0	9,1	8,0	6,9	7,7	2,6	9,3	6,9	5,5	0	9,2	9,0
LVAD power (Watt)	3.4	5,0	4,0	3,9	4,6	3,7	3,3	2,8	4,4	4,4	3,5	1,8	5,4	4,9
LVAD speed (rpm)	2400	2300	2360	2340	2400	2560	2360	2480	2500	2400	2400	2400	2500	2400
**Post intervention**														
LVAD speed (L/min)	4,6	3,7	2,8	4,0	3,0	5,2	5,4	9,5	4,1	3,0	3,6	3,2	4,7	3,4
LVAD power (Watt)	3,0	2,7	2,8	2,8	3,0	3,5	3,1	5	3,8	3,1	3,0	2,9	3,7	3,0
LVAD speed (rpm)	2400	2300	2360	2340	2400	2560	2360	2480	2500	2400	2400	2400	2500	2400

Risk factors for pump thrombosis and laboratory values at the time of pump thrombosis. Moreover, LVAD flow, power and speed values at baseline, during systemic thrombolysis and post intervention. The flow in patient 8 remained high post intervention because of persistent pump thrombosis. The flow pre-intervention in patient 12 was 0 L/min (for several short times).

INR, international normalized ratio; LDH, lactate dehydrogenase; LVAD, left ventricular assist device; MAP, mean arterial pressure; N/A, not applicable; OAC, oral anticoagulation; PT, pump thrombosis.

Table 3 shows that systemic thrombolysis was successful in 10 of 14 patients (71%) at 30 days. Two of the 14 patients (14%) died within 30 days after an in hospital cardiac arrest due to a hemothorax (patient 7), and multi-organ failure (patient 8, as described above), respectively. In patient 7, systemic thrombolysis was considered the only suitable option despite the recent LVAD implantation. Unfortunately, this resulted in an early fatal bleeding. Three patients experienced a thrombotic complication within 30 days; all recurrent PT. Patient 8 died as a result of multi-organ failure (received 3 times systemic thrombolysis). Patient 13 and 14 received thrombolysis again, respectively 5 and 4 days after their first systemic thrombolysis. Patient 13 was free of PT thereafter and patient 14 experienced 3 additional events of pump thrombosis. Six patients had a 30-day bleeding complication of which 3 major bleedings according to the ISTH criteria (twice a hemothorax (one fatal ending) and one right calf bleeding, no intracerebral hemorrhage). No patient experienced an intracerebral hemorrhage after systemic thrombolysis. Eleven of the 14 DT patients were discharged home shortly after thrombolysis (average of 11 days).

**Table 3:** Treatment success and 30-day outcomes.

**Patient no**.	**Treatment** ** success**	**30-day** ** mortality**	**30-day thrombosis** ** complications**	**30-day bleeding** ** complications**	**Time between** ** thrombolysis and** ** discharge (days)**	**Time between PT** ** and mortality or** ** latest follow-up (days)**
1	Yes	No	No	No	5	1830
2	Yes	No	No	No	28	850
3	Yes	No	No	No	4	216
4	Yes	No	No	Hemothorax	41	862
5	Yes	No	No	Right calf bleeding	9	904
6	Yes	No	No	Psoas bleeding	N/A	135
7	No	Yes	No	Hemothorax	N/A	0
8	No	Yes	Recurrent PT	No	N/A	6
9	Yes	No	No	No	6	415
10	Yes	No	No	No	6	114
11	Yes	No	No	No	3	263
12	Yes	No	No	No	6	499
13	No	No	Recurrent PT	Epistaxis	11	129
14	No	No	Recurrent PT	Blood seeping from wound treated by VAC therapy	4	40
Total	71%	14%	21%	3 major bleedings (21%)	N/A	N/A

Treatment success, and 30-day mortality, thrombosis and bleeding complications after systemic thrombolysis are shown. Moreover, times between PT latest follow-up and -discharge and times between PT and mortality are depicted. Patient 14 has been discharged at day 4 following systemic thrombolysis. Flow and power returned to baseline levels. However, within 30 days he presented with a recurrent pump thrombosis and therefore there is no treatment success as defined by “patient alive at 30 days follow-up and free of recurrent PT, stroke or device exchange”. He underwent recent reversal of his oral anticoagulation because of surgical drainage and VAC placement because of a driveline infection.

N/A, not applicable; PT, pump thrombosis; VAC, vacuum assisted closure.

After PT, no changes in regular medical regimen of clopidogrel/phenprocoumon were implemented in nine patients but often INR target range was revised. In three patients a switch to prasugrel was made because of inadequate inhibition of ADP-induced aggregation (i.e., clopidogrel resistance). In the remaining two patients nadroparin monotherapy was used awaiting the recovery of bleeding in the calf, and prasugrel/heparin was used awaiting the recovery of a hemothorax.

## Discussion

The main finding of the current study is that systemic thrombolysis for PT is feasible and successful in the majority of the cases regarding resolution of PT in DT LVAD patients. Complications were often well manageable and must be interpreted in the light of no reasonable alternative treatment options for this life-threatening event in this vulnerable patient population.

Several studies reported on the feasibility and safety of medical therapy for PT in LVAD patients (6, 8, 9), and compared this with surgical device exchange (10). Medical treatment strategies for pump thrombosis vary among different centers. Direct thrombin inhibitors, tissue plasminogen activator, or glycoprotein IIb/IIIa antagonist have been reported as options (7).

In the literature, two studies reported the use of alteplase as initial and single medical treatment (not combined with other anticoagulant modalities) in the light of PT in patients with a HeartWare device. First, there is a case report from Kamouh et al. (8) in which they treated a patient successfully with a total dose of 30 mg alteplase (with a successful transplant 30 days after thrombolytic therapy). Second, there is another case report of Heim et al. (11) describing a case where they used 50 mg alteplase iv in total (20 mg over 60 minutes and the remaining 30 mg over the next 3 h thereafter). In conclusion, medical treatment options in general are heterogeneous and even within the group who receive tissue plasminogen activator it is mixed.

A recent meta-analysis of 43 studies comprising 28.728 LVAD patients suggests that surgical device exchange is superior to medical therapy for PT (7). This meta-analysis should, however, be regarded as indirect evidence as no randomized trial comparing medical therapy with pump exchange has been performed. Also, this meta-analysis included the whole LVAD population whereas we specifically focused on DT LVAD patients. Treatment success has previously been defined as patients alive and free from recurrent PT, stroke, device exchange or urgent transplantation at 30 days follow-up (6, 7). Although there is experience with systemic thrombolysis for the treatment of PT, the regimen used varies among different centers and success rate depends on early recognition of PT (12). In our study, medical therapy, using a structured thrombolysis protocol, was successful in 10 of 14 patients (71%) at 30 days, whereas the recent meta-analysis reported a much lower success rate of 45% for medical therapy (7). Moreover, the current 14% 30-day mortality rate compares favorably to the previously reported 17% mortality rate of surgical therapy (7).

The current findings are of particular relevance given the recent withdrawal of the HeartWare LVAD system of the market by Medtronic (13). A substantial number of the 4000 living patients have their HeartWare LVAD as *DT*, and exchange to a HeartMate 3 may be challenging in this vulnerable patient population (12). The exchange from HeartWare LVAD to a HeartMate 3 is surgically and technically feasible, but the surgery is more complex than just a regular exchange from HeartWare to HeartWare and the redo surgery *per se* is associated with increased post-operative risks and mortality. Therefore, medical treatment using systemic thrombolysis may be a viable option in this patient group with a PT as supported by our results. To our knowledge, there has no study been performed comparing systemic thrombolysis with exchange from HeartWare LVAD to HeartMate 3.

Some limitations of this study need to be addressed. First, the small sample size with only DT patients and single center design might hamper the extrapolation of our data to *all* LVAD patients with a HeartWare LVAD. In the bridge-to-transplant or bridge-to-recovery cohort the occurrence of PT unresponsive to medical therapy can lead to urgent transplantation but in our DT group this is not an option. Second, the observational, retrospective study design does not allow for firm conclusions regarding the efficacy of thrombolysis to manage PT in DT LVAD patients. Still, adherence to a structured management protocol during the course of this study and complete data collection, strengthen the validity of our observations.

## Conclusion

Systemic thrombolysis may be a reasonable therapeutic option in DT LVAD patients with a PT as final attempt to prevent death or life-threatening events, despite its risk of bleeding complications. The decision to give this therapy should be made in a multidisciplinary team with the patient and his/her family, thrombosis and hemostasis experts, cardiothoracic surgeons and cardiologists.

## Data availability statement

The raw data supporting the conclusions of this article will be made available by the authors, without undue reservation.

## Ethics statement

The studies involving human participants were reviewed and approved by the Leiden University Medical Center, Leiden, The Netherlands. Written informed consent for participation was not required for this study in accordance with the national legislation and the institutional requirements.

## Author contributions

KK was responsible for conceptualization, investigation, analysis, editing, and writing. PdE and LT were responsible for conceptualization, investigation, analysis, writing, and supervision. SB, MP, JJ, and MH were responsible for conceptualization and supervision. All authors contributed to the article and approved the submitted version.

## Conflict of interest

The authors declare that the research was conducted in the absence of any commercial or financial relationships that could be construed as a potential conflict of interest.

## Publisher's note

All claims expressed in this article are solely those of the authors and do not necessarily represent those of their affiliated organizations, or those of the publisher, the editors and the reviewers. Any product that may be evaluated in this article, or claim that may be made by its manufacturer, is not guaranteed or endorsed by the publisher.

## References

[1] Potapov EV Antonides C Crespo-Leiro MG Combes A Farber G Hannan MM. 2019 EACTS Expert Consensus on long-term mechanical circulatory support. Eur J Cardiothorac Surg. (2019) 56:230–70. 10.1093/ejcts/ezz098 31100109 PMC6640909

[2] Chou BP Lamba HK Cheema FH Civitello AB Delgado RM Simpson L. Outcomes of repeat left ventricular assist device exchange. ASAIO J. (2020) 66:64–8. 10.1097/MAT.0000000000000928 30507849

[3] Webber BT Panos AL Rodriguez-Blanco YF. Intravenous thrombolytic therapy for patients with ventricular assist device thrombosis: an attempt to avoid reoperation. Ann Card Anaesth. (2016) 19:192–6. 10.4103/0971-9784.173047 26750701 PMC4900393

[4] Kormos RL Antonides CFJ Goldstein DJ Cowger JA Starling RC Kirklin JK. Updated definitions of adverse events for trials and registries of mechanical circulatory support: a consensus statement of the mechanical circulatory support academic research consortium. J Heart Lung Transplant. (2020) 39:735–50. 10.1016/j.healun.2020.03.010 32386998

[5] Schulman S. Kearon C. Definition of major bleeding in clinical investigations of antihemostatic medicinal products in non-surgical patients. J Thromb Haemost. (2005) 3:692–4. 10.1111/j.1538-7836.2005.01204.x 15842354

[6] Stulak JM Dunlay SM Sharma S Haglund NA Davis MB Cowger J. Treatment of device thrombus in the HeartWare HAVD: success and outcomes depend significantly on the initial treatment strategy. J Heart Lung Transplant. (2015) 34:1535–41. 10.1016/j.healun.2015.10.020 26681123

[7] Luc JGY Tchantchaleishvili V Phan K Dunlay SM. Maltais S, Stulak JM. Medical therapy as compared to surgical device exchange for left ventricular assist device thrombosis: a systematic review and meta-analysis. ASAIO J. (2019) 65:307–17. 10.1097/MAT.0000000000000833 29863631

[8] Kamouh A John R Eckman P. Successful treatment of early thrombosis of HeartWare left ventricular assist device with intraventricular thrombolytics. Ann Thorac Surg. (2012) 944:281–3. 10.1016/j.athoracsur.2011.12.024 22734996

[9] Bitar A Vijayakrishnan R Lenneman A Birks E Massey T Slaughter M. The use of eptifibatide alone or in combination with heparin or argatroban for suspected thrombosis in patients with left ventricular assist devices. Artif Organs. (2017) 41:1092–8. 10.1111/aor.12910 28621461

[10] Oezpeker C Zittermann A Ensminger S Kizner L Koster A Sayin A. Systemic thrombolysis vs. device exchange for pump thrombosis management: a single-center experience. ASAIO J. (2016) 62:246–51. 10.1097/MAT.0000000000000340 26771393

[11] Heim C Kondruweit M Weynan M Tandler R. Amphetamine abuse as a rare cause of recurrent LVAD pump thrombosis. J Card Surg. (2015) 30:215–6. 10.1111/jocs.12504 25545600

[12] Hayward C Adachi I Baudart S Davis E Feller ED Kinugawa K. Global best practices consensus: Long-term management of patients with hybrid centrifugal flow left ventricular assist device support. J Thorac Cardiovasc Surg. (2022) 164:1120–37. 10.1016/j.jtcvs.2022.03.035 35624053

[13] Tops LF Coats AJS Ben Gal T. The ever-changing field of mechanical circulatory support: new challenges at the advent of the 'single device era.' Eur J Heart Fail. (2021) 23:1428–31. 10.1002/ejhf.2314 34296495 PMC9290740

